# Smart sensors in Thai dairy reproduction: A case study

**DOI:** 10.14202/vetworld.2024.1251-1258

**Published:** 2024-06-08

**Authors:** Jirayus Kaewbang, Jidapa Lohanawakul, Napat Ketnuam, Kachapas Prapakornmano, Pongsanan Khamta, Aqeel Raza, Theerawat Swangchan-Uthai, Davids Makararpong, Chaidate Inchaisri

**Affiliations:** 1 Research Unit of Data Innovation for Livestock, Department of Veterinary Medicine, Faculty of Veterinary Science, Chulalongkorn University, 10330 Bangkok, Thailand; 2 Chulalongkorn Animal Hospital, Faculty of Veterinary Science, Chulalongkorn University, 73000 Nakhonpathom Province, Thailand; 3 International Graduate Program of Veterinary Science and Technology, Faculty of Veterinary Science, Chulalongkorn University, 10330 Bangkok, Thailand; 4 CU-Animal Fertility Research Unit, Department of Veterinary Obstetrics and Gynecology, Faculty of Veterinary Science, Chulalongkorn University, 10330 Bangkok, Thailand; 5 Senovate AI Co., Ltd., 10240 Bangkok, Thailand

**Keywords:** dairy cattle, movement activity sensors, reproductive performance

## Abstract

**Background and Aim:**

Movement activity sensors are known for their potential to boost the reproductive performance of dairy cows. This study evaluated the effectiveness of these sensors on three Thai dairy farms (MK, NF, and CC), each using different sensor brands. We focused on reproductive performance at these farms and expanded our evaluation to include farmer satisfaction with sensor technology on five farms (MK, NF, CC, AP, and IP), allowing for a thorough analysis of both operational outcomes and user feedback.

**Materials and Methods:**

A total of 298 lactation records and interviewing five experienced farm owners with over a year of sensor usage were our research methods. To measure the effect on the first service timing and post-parturition pregnancy rates, Cox regression models were utilized for sensor usage.

**Results:**

Biosensors’ implementation enhanced data precision while quickening the first service within 100 days and pregnancy within 200 days. The MK and NF farms showed significant progress. Within 100 and 200 days post-implementation, the overall improvement was 30%–34% in the first service rate and 39%–67% in the conception rate across all assessed farms. Farmers acknowledged improved reproductive performance from the sensors, overcoming language barriers.

**Conclusion:**

The study highlighted the advantages of using movement activity sensors in enhancing both cattle reproductive success and farmers’ satisfaction on Thai dairy farms. These sensors led to more accurate management decisions, increasing overall farm productivity.

## Introduction

Maximizing dairy cow productivity and longevity, as well as profitable breeding, relies heavily on reproductive fertility [1, 2]. Delayed onset of lactation decreases overall production efficiency due to a longer lactation period with a lower milk yield [3, 4]. Estrus behavior’s intensity significantly impacts reproductive success [5–9]. Effective and precise detection of estrus in dairy cows remains an ongoing concern [10].

Estrus detection mainly depends on visual observations of behavioral shifts such as standing, additional walking, and restlessness [11, 12]. These methods necessitate frequent labor-intensive observations, performed by skilled and experienced personnel, 2–5 times a day [13, 14]. High-yielding dairy cows demonstrate less intense and shorter estrus behaviors, complicating identification through visual observation [13, 15]. While teaser bulls serve as an alternative method to traditional ways of detecting estrus in dairy cattle [16–19]. The effectiveness of identifying cattle in estrus through teaser bulls can be compromised by their reduced sensitivity, leading to missed breeding opportunities [10, 17, 18]. Integrating teaser bulls into dairy farm operations brings additional costs for feeding and operations as well as increased logistical complexity [17, 18]. Animal welfare regulations and potential injury risks may completely prohibit the use of teaser bulls [16].

Automated monitoring systems offer a promising solution by enabling continuous and real-time identification of individual animals, reducing labor requirements, and providing estimates of ovulation timing [20]. Triaxial accelerometers measure dynamic and static accelerations induced by an animal’s movement, generating quantifiable output waveforms [21]. These devices can analyze and classify animal postures based on their activity and movement patterns [22]. Real-time data collection and automated data transmission to base stations facilitated continuous monitoring [23]. A previous study reported that this technological intervention can enhance estrus detection accuracy by up to 80%–90%, surpassing the capabilities of traditional methods [24]. By facilitating breeding at the optimal time for conception, biosensor technology can significantly improve reproductive performance [25], contributing to Thai dairy farms’ overall economic sustainability.

The implementation of smart biosensor devices in Thai dairy farms faces challenges due to potential dairy cattle behavior changes, conflicting farm management practices, and labor skill shortages. Approximately 100 Thai dairy farms currently use imported advanced devices. The effectiveness of activity-monitoring sensors for improving reproductive fertility is yet to be demonstrated on Thai dairy farms. This study aimed to fill the knowledge gap regarding the effect of movement sensors on reproductive performance and farmer satisfaction in Thai dairy farms. This study highlights the benefits and drawbacks of implementing activity monitoring systems in Thai dairy farms. The findings will significantly impact future dairy industry implementation strategies in Thailand, promoting sustainable and efficient dairy production.

## Materials and Methods

### Ethical approval and Informed consent

This field study, focusing on data gathered directly from farm databases, did not involve direct interaction with animals but analyzed existing data related to their reproductive performance. Consequently, ethical approval from an institutional review board was not required for this aspect of the research.

Prior to data collection, informed consent was obtained from all participating farm owners. They were thoroughly informed about the objectives of the study, the nature of their involvement, and the use of data from sensor technology on their animals. Consent was also secured for accessing their farm databases to gather the necessary data for the study.

All procedures were designed to ensure the confidentiality and anonymity of the farm owners and their operational data, upholding ethical standards and protecting participant privacy throughout the research process.

### Study period and location

Data collection took place from April to July 2022 on farms located in Saraburi Province in the central part of Thailand, a key area for dairy production. The research on reproductive performance after implementing sensor technology was conducted on three dairy farms: MK, NF, and CC. Additionally, to assess farmer satisfaction with the use of sensor technology, the study included two more farms, AP and IP, making a total of five farms (MK, NF, CC, AP, and IP) involved in this aspect of the research. This setup provided a comprehensive overview of both the quantitative effects on animal health and qualitative feedback from farm owners about the technology’s benefits.

### Data collection

#### Reproductive management

At dairy farms such as MK, NF, and CC, veterinary-led initiatives have integrated smart biosensors into their reproductive management practices. Based on sensor data, veterinary professionals optimized reproductive management strategies on MK, NF, and CC dairy farms through on-site visits, offering direct support.

#### Activity biosensors

An accelerometer within a non-invasive wearable biosensor system on dairy cattle gathers raw data. The built-in algorithms analyze raw data to determine activity patterns, step count, and duration of eating, rumination, and lying. These devices for monitoring dairy cow activity are categorized according to their on-body locations, such as pedometers, neck collars, reticulum-rumen boluses, or ear tags [22, 26]. Ear tags and neck collars were used in this study to gather essential data. Device selection depended on particular factors and the appropriateness of the sites for data collection.

#### Reproductive performance index

The study employed activity sensors from three Thai dairy farms (MK, NF, and CC) to collect data on lactating dairy cattle. A total of 298 lactation records from three different dairy farms were evaluated to measure the effect of smart biosensors on reproductive performance. Seventy records were from the CC dairy farm, 134 from the MK dairy farm, and 94 from the NF dairy farm. Lactations ranged from first to third or beyond. Each dairy farm used a distinct activity sensor brand. This study benefited from the accessibility of pre-implementation reproductive data from both the MK and NF dairies. Analyzing reproductive metrics before and after biosensor implementation on these dairy farms allowed for a valuable comparison. Sixty-seven lactation records per period from MK dairy farm were available for analysis compared to data from NF dairy farm for 24 lactations before and 70 lactations after biosensor adoption. The study’s design, which includes a pre- and post-implementation phase, enhances the study’s internal validity and offers a more complex perspective on the effects of the technology.

This study examined the relationships between dairy cow reproductive performance, lactation number, and on-farm software data, particularly focusing on the calving-to-first-service and calving-to-conception intervals. Data from three dairy farms (MK, NF, and CC) were classified according to the lactation stage (first, second, or third). At MK and NF farms, analysis was conducted to evaluate the impact of implementing movement sensors before and after. CC was established with movement sensors. Cows calving at least 100 days before biosensor implementation were classified as “before.” The subsequent analyses utilized data from all three farms. The heat detection rate and reproductive efficiency were determined based on the comparison of the average intervals between actual and expected estrous cycles. The study sought to illuminate the impact of lactation number, biosensor technology usage, and unique farm management on dairy cow reproductive success using farm software data.

#### Satisfaction survey and opinion gathering

A qualitative study using in-depth interviews was conducted to explore farmers’ perceptions and the actual effect of biosensor technology on dairy production. Five dairy farms were strategically chosen, including three farms (MK, NF, and CC) that had been previously studied for technology’s impact on animal reproduction, and two other farms that declined to disclose extensive data in a prior investigation. Ensuring diverse perspectives from both adopters and non-adopters was accomplished. Farm owners with over a year of experience using biosensors provided informed and valuable insights during the study. This study sought to deepen our knowledge of dairy farmers’ subjective experiences, perceived benefits, and difficulties in using biosensor technologies. Farm owners articulated the pros and cons of incorporating biosensors into their businesses through one-on-one interviews. By combining essential topics such as data acquisition and utilization, owner satisfaction, reproductive management practices, farm management evolution, potential labor efficiencies, and interpersonal dynamics within the farm workforce, we designed a carefully structured interview schedule for an in-depth and productive conversation. The study expanded on the qualitative findings by investigating the implementation of biosensors on dairy farms. The interview questions were thoroughly examined.

### Statistical analysis

Data analysis was carried out through Statistical Package for the Social Sciences software version 22 (IBM Corp., Armonk, NY, USA). Initial exploratory analyses were carried out to determine the factors impacting calving-to-first-service interval (CFSI) and calving-to-conception interval (CCI). Cox regression models were used to estimate the hazard ratios (HRs) for reaching the first service by 100 days in milk (DIM) and pregnancy by 200 DIM. At farms MK and NF, the temporal effects before and after biosensor implementation were analyzed during the first study. This second analysis scrutinized farm-level disparities in reproductive performance by analyzing the data collectively from all three farms.

Using Cox regression models, we comprehensively understood the factors influencing reproductive performance by incorporating several exploratory variables. The farm identification (CC, NF, or MK) was incorporated to adjust for any distinct farm influences. 1, 2, and ≥3 categorized lactation numbers to reduce confounding variable effects related to the lactation stage. Lactation No. 1 is set as the reference category in this analysis. The independent effect of lactation number on study outcomes was assessed clearly using this approach. The effects of biosensor implementation were compared between farms MK and NF, using the pre-implementation period as a reference.

Cows that were conceived before 200 DIM or inseminated before 100 DIM had their observations censored at DIM, whereas those not inseminated by 100 DIM had theirs censored at 100 DIM. Cows conceiving after day 200 were censored at 200 DIM. About 95% confidence intervals (CIs) and p-values accompanied the reported adjusted HRs. The statistical significance of findings in the Cox regression models was determined using a 95% CI and a p-value threshold of < 0.05.

## Results

### Reproductive performance analysis

A total of 298 lactation records were analyzed for reproductive performance evaluations. Of 207 records from the lactation groups, post-biosensor implementation was available for analysis: lactation No. 1 (n = 86, 41.15%), lactation No. 2 (n = 77, 37.2%), and lactation No. ≥3 (n = 44, 21.3%). Additional farm-level categorizations revealed 70 records from the CC farm, 67 from the MK farm, and 70 from the NF farm.

After adopting biosensors, there was a notable enhancement in heat detection rates. At MK and NF farms, the pre-implementation rates were 22.8% and 10.5%, respectively. The farms experienced significant improvements in heat detection post-implementation: MK (44.2%), NF (66.7%), and CC (40.5%). The accuracy of heat detection can be significantly enhanced through the use of biosensor technology, thereby impacting overall reproductive performance.

In the analysis of the CFSI using cumulative survival curves, a statistically significant difference (p < 0.05) was found between the pre- and post-biosensor groups, necessitating approximately 40 extra days for farm MK to achieve a 34% enhancement in the CFSI rate over the post-biosensor group. At the NF dairy farm, it took an extra 30 days to reach a 30% enhancement in the CFSI rate without the biosensor technology. The adoption of biosensors on farms resulted in a significant improvement in reaching the first service.

In Table-1, the impact of lactation numbers (one, two, and three or more) on the success of first insemination within 100 days in milk is displayed at MK and NF farms. At MK farm, survival rates significantly varied between lactation groups at the 100-DIM mark. Cows in their second lactation had a significantly higher likelihood of achieving first insemination at 100 DIM, with an HR of 1.97 (95% CI: 0.99–3.98, p < 0.05), as opposed to those in their first lactation. The differences between cows in their third or subsequent lactations and those in their first lactation were insignificant. At the NF farm, no significant differences in success rates were noted between lactation groups. At MK farm, the second lactation has the highest success rate for first insemination within 100 DIM.

**Table-1:** The Cox regression model was applied to analyze the CFSI within 100 DIM and the CCI within 200 DIM in relation to lactation number (Lact. No.) and the implementation of sensor technology at MK and NF farm.

Parameters	Variables	n	β	HR	p-value	95% CI for HR	

						Lower	Upper
MK farm							
CFSI within 100 DIM	Lact. No. 1	60	Ref.				
	Lact. No. 2	38	0.67	1.97	0.05	0.99	3.89
	Lact. No. ≥3	36	0.41	1.51	0.27	0.72	3.13
	Before using sensor	67	Ref.				
	After using sensor	67	1.14	3.12	< 0.01	1.64	5.95
CCI within 200 DIM	Lact. No. 1	60	Ref.				
	Lact. No. 2	38	0.20	1.22	0.62	0.57	2.62
	Lact. No. ≥3	36	−0.19	0.83	0.69	0.33	2.08
	Before using sensor	67	Ref.				
	After using sensor	67	1.67	5.04	< 0.01	2.32	12.21
NF farm							
CFSI within 100 DIM	Lact. No. 1	31	Ref.				
	Lact. No. 2	31	0.40	1.49	0.24	0.77	2.87
	Lact. No. ≥3	32	−0.34	0.71	0.37	0.34	1.49
	Before using sensor	24	Ref.				
	After using sensor	70	0.8	2.23	0.02	1.14	4.37
CCI within 200 DIM	Lact. No. 1	31	Ref.				
	Lact. No. 2	31	0.17	1.18	0.71	0.49	2.87
	Lact. No. ≥3	32	−0.82	0.44	0.15	0.15	1.33
	Before using sensor	24	Ref.				
	After using sensor	70	2.83	16.95	< 0.01	3.7	76.92

DIM=Days in milk, CFSI=Calving to first service interval, CCI=Calving to conception interval, HR=Hazard ratio, CI=Confidence interval

At MK and NF farms, CCI analysis with Cox regression and survival curves showed significant differences (p < 0.01) between groups before and after biosensor implementation. At 200 DIM, the biosensor technology led to substantially higher conception rates at both farms, yet the improvement was more noticeable at the NF farm. After implementing biosensors, MK Farm’s pregnancy rate increased by a factor of five (CCI HR: 5.32, p < 0.01). The percentage increase from pre- to post-biosensor stages rose significantly, from 11% to 50%. The increase in CCI HR from 8% to 75% at the NF farm was statistically significant (p < 0.02), with a value of 16.95. These findings reveal biosensor technology’s significant capacity to enhance conception rates in dairy farming, despite lactation number having no substantial effect on conception success at 200 DIM on both farms. Factors other than lactation appear to be more critical than lactation in determining pregnancy success on MK and NF farms within this timeframe.

Using a Cox regression model (Table-2), factors affecting the CFSI and CCI were identified post-biosensor implementation in all three farms. In the analysis, MK farms had lower rates of both CFSI and CCI than NF farms; yet, lactation numbers did not significantly impact the findings. At the MK farm, conception rates were 50% lower than at the NF farm, which boasted a 75% success rate. This disparity in early pregnancy achievement among farms warrants investigation into context-specific influencing factors.

**Table-2:** The Cox regression model was utilized to assess the CFSI within 100 DIM and the CCI within 200 DIM following the implementation of movement activity sensors across three farms.

Parameters	n	β	HR	p-value	95% CI for HR	

					Lower	Upper
CFSI within 100 DIM						
Lact. No. 1	86	Ref.				
Lact. No. 2	77	–0.01	0.99	0.99	0.65	1.53
Lact. No. ≥3	44	–0.42	0.66	0.15	0.37	1.17
Farm NF	70	Ref.				
Farm CC	70	–0.04	0.96	0.79	0.72	1.28
Farm MK	67	–0.33	0.72	0.01	0.55	0.94
CCI within 200 DIM						
Lact. No. 1	86	Ref.				
Lact. No. 2	77	0.01	1.01	0.98	0.57	1.77
Lact. No. ≥3	44	–0.64	0.53	0.09	0.25	1.11
Farm NF	70	Ref.				
Farm CC	70	–0.17	0.85	0.39	0.57	1.24
Farm MK	67	–0.33	0.72	0.05	0.53	1.0

DIM=Days in milk, CFSI=Calving to first service interval, CCI=Calving to conception interval, HR=Hazard ratio, CI=Confidence interval

### Satisfaction

AP farm personnel interviewed revealed both the advantages and challenges of biosensor technology implementation. Improved heat detection accuracy resulted in more timely inseminations and possibly enhanced reproductive performance. Biosensors that can detect sick cows through decreased activity levels enable early intervention, potentially improving health outcomes. Users appreciated the prompt and convenient notifications in the mobile app for making quick responses to cow behavior and health changes.

User needs and limitations were acknowledged. The effectiveness of biosensors in identifying early estrus could affect overall heat detection accuracy. Frequent manual adjustments because of biosensor sensitivity issues underscored the importance of user-friendly features and automated calibration. The absence of a Thai language option in the mobile app restricts some workers from fully utilizing it.

The owner of MK farm commended the biosensor technology while acknowledging its implementation hurdles. The biosensors’ precise heat detection was highly appreciated by the owner, ensuring timely insemination of cows in estrus. This capability has enhanced farm conception rates and overall reproductive performance. The real-time notification system’s value lies in enabling prompt breeding decisions based on real-time estrus information.

The MK farm, though beneficial, faces hurdles in biosensor technology. The absence of a Thai language option for cell phone apps has negatively impacted workers’ understanding and efficiency in using the system. The owner mentioned frequent loss of ear-tag sensors, implying they may have been dislodged from the cows. The reliability of data collection and overall worker comprehension of technology have become major concerns. To enhance stability and maintain data consistency, the owner proposed testing neck collars as an alternative placement for the biosensor.

The owner of the NF Farm shared positive experiences and identified challenges related to implementing biosensors during the interview. Effectively removing the language barrier, Thai language support for cell phone applications was highly valued among dairy farm personnel. This factor significantly impacts technology utilization and employee involvement. Due to the biosensors’ accurate heat detection and subsequent timely insemination, the owner noted a noteworthy enhancement in cattle conception rates. Determining a cow’s illness by recognizing decreased activity levels is essential for timely intervention and better health results. Owners raised several issues. The Internet connection between the biosensors and the central system sometimes caused interruptions. A critical factor for maximizing the advantages of biosensor technology in dairy farm management and improvement is identified as data transmission stability.

During an interview at CC Farm, a farmer veterinarian shared insights on the benefits and challenges of implementing biosensor technology. The veterinarian commended the biosensors for their efficient identification of potential heat events in cows. By specifically focusing on heat detection, this approach speeds up the process and enhances overall workflow productivity. The application’s functionality and responsiveness enhanced daily farm operations and improved management effectiveness.

Despite improvements in operational efficiency, heat detection accuracy and overall conception rates did not significantly improve. To achieve substantial improvements in reproductive performance, more optimization and calibration of the farm management technology system may be necessary. The biosensor signal was initially unstable, requiring its reinstallation. A seamless setup process is crucial for biosensor technology’s successful and dependable implementation on dairy farms.

Through an interview with the IP farm owner, advantages and challenges of biosensor technology implementation were disclosed. The technology’s ability to significantly reduce labor requirements was acknowledged by the owner, leading to time savings and reduced economic costs. Enhanced operational efficiency leads to increased farm profits. The biosensor data’s accuracy pleased the owner, enhancing decision-making processes and contributing to the herd’s reproductive success.

Despite its benefits, the use of this advanced biosensor technology comes with drawbacks. A higher initial purchase cost may deter smaller dairy farms, especially those without external financing options. The owner emphasized the importance of Thai language support in mobile apps. The absence of native language support restricted the dairy farm team’s accessibility and ease of use, emphasizing the significance of linguistically accommodating interfaces.

## Discussion

Variations in contraceptive effectiveness corresponding to pregnancies between 90 and 150 days after implantation were studied, focusing on potential changes within the usual ideal window of 12–14 months [27]. Significant differences in conception rates were observed between MK and NF farms, despite both falling within the recommended optimal range. Conception rates within 200 DIM before the implementation of movement sensors were lower compared to previous studies. This finding contrasts with those reported in the literature, such as those conducted by Vargas *et al*. [28] in Costa Rica, where rates ranged from 75% to 88%, and by Temesgen *et al*. [29] in Ethiopia, where rates were between 62% and 90%. In addition, studies conducted in other regions, like the one by Kim and Jeong [30] in Korea, reported rates of 55%–95% at 210 DIM, while Khemarach *et al*. [31] noted a 70% rate at 160 DIM, and Kornmatitsuk *et al*. [32] found rates of 33%–62% at 150 DIM in Thailand. The regional and farm-specific factors significantly impact conception rates, as evidenced by these variations. The complex interplay of factors at both the herd and lactation levels may account for variations in the CCI between farms and regions. Potential contributors to this variability include inadequate heat detection by farmers after calving, leading to delayed insemination [29], the influence of external factors such as season [33], environmental conditions (temperature-humidity index), and calving-related complications such as dystocia, which can impact the calving-to-insemination interval and subsequently prolong CCI [30].

The biosensor detection showed a significant variation in CCI between the MK and NF farms. Reproductive efficiency at farm MK improved from 11% to 50%. The NF farm showed a remarkable increase in the CCI, rising from 8% to 75%. These substantial interfarm differences suggest a robust potential for biosensors to optimize conception rates, particularly when traditional heat detection practices are suboptimal [20, 34]. In this study, the number of previous lactations (1, 2, or ≥3) did not affect the conception rates at either farm. In some contexts, the biosensor’s efficacy in detecting estrus is amplified by pre-existing challenges in NF estrus detection or herd-specific characteristics. More investigations are needed to clarify the exact reasons for these differences.

CFSI and accurate heat detection significantly determine CCI in dairy cattle [35]. CFSI faced less interest as a reproductive measurement due to its measurement challenges compared to heat detection [35]. The strong correlation between CFSI and calving interval reinforces the significance of optimizing CFSI to achieve a calving interval of between 12 and 14 months [36, 37]. This study identified a statistically significant difference (p < 0.05) between CFSI levels pre- and post-biosensor implementation for both MK and NF farms. 30%–34% increase in reaching the first service was achieved by cows with biosensors in the first 100 DIM. Smart biosensors effectively identify heat periods, reducing CFSI and optimizing dairy herd reproduction cycles. Previous studies by Vargas *et al*. [28], Temesgen *et al*. [29], Francos and Mayer [38], and Thompson *et al*. [39] also reported higher pregnancy rates in dairy cows with shorter calving-to-insemination intervals.

Using data from both MK and NK farms, this study’s findings indicate that activity biosensors can significantly improve reproductive success in dairy cattle. The heat decision rate increase and better CFSI/CCI results post-sensor implementation corroborate this hypothesis. Previous research by Kim *et al*. [40] supports the positive correlation between lactation number and CFSI in MK dairy farms. Madureira *et al*. [5] and Tippenhauer *et al*. [41] present contrasting findings. Future studies must account for farm-specific factors to draw consistent and comprehensive comparisons of pre- and post-biosensor implementation results.

According to Reith and Hoy [42], the use of smart devices and monitoring systems greatly improves estrus detection over traditional visual observation. The achieved heat detection rate still falls short of the recommended target range of 65%–70% [43]. This discrepancy might be caused by factors such as the impact of other cows and the effects of environmental conditions on the health of individual cows. In tropical climates with high temperatures and humidity, negative energy balance and heat stress are associated with anestrus and decreased fertility rate [41]. To optimize reproductive outcomes in dairy farms, it is necessary to conduct more research on the relationship between environmental factors and biosensor efficacy.

It is plausible that these farms, with below-par reproductive performance, opted for movement sensor technology. These investments notably improve farm productivity, specifically in relation to reproductive efficiency. Before implementing movement sensors, lower conception rates were observed, which is consistent with this explanation. Adopting new technologies such as movement sensors and farms may prevent and improve reproductive issues proactively. Understanding why these farms adopted advanced monitoring tools lies in the perspective that they aimed to enhance reproductive outcomes.

In line with prior research, participants in our study highlighted substantial time and labor reduction. The interface presented a major language barrier issue in this study. Recognizing the significance of cultural context and dairy farmers’ needs is crucial in effectively designing and deploying smart biosensors. The incomprehensible English interface could damage owner trust and engagement with the smart-sensing technology. Overcoming language barriers is essential for providing equitable access and amplifying the value of sensing technologies to all dairy farmers. This study proposes multilingual junctions, targeted training programs, or local communities with trained veterinarians as potential solutions. Studies were needed to explore the influence of language barriers and cultural factors on smart sensing technology adoption and dairy farmers’ satisfaction in varied agricultural contexts. Based on the study’s results, mobile applications tailored to the dairy farming culture and user experience can enhance their adoption and social acceptance within the dairy farming community.

The small sample size of dairy farms used in this study limits its applicability to the larger Thai dairy farming community. The analysis was restricted by the limited participation (only five dairy farm owners) in the study. Some farmers voice fears regarding data privacy and ownership with the integration of biosensors and smart sensing technology. Addressing data security concerns and building trust among dairy farmers was essential for their engagement in data-driven research initiatives. Implementing data privacy and security frameworks, as proposed in some countries [44], in Thailand would necessitate the cooperation and compliance of all stakeholders. The substantial costs and legal complexities of extensive data collection and agreements pose major challenges. Some farmers may be reluctant to adopt advanced reproductive management technologies due to cultural and ethical beliefs, as well as concerns about the complexity of the technology. To overcome these research limitations, future studies should propose new data collection and sharing methods, provide cost-effective behavioral monitoring solutions, and examine Thai dairy farmers’ cultural influences on technology adoption. Addressing the limitations and fostering trust in future research will broaden the use of smart-sensing technologies in the dairy farming industry.

## Conclusion

Significant improvements in reproductive performance, including notable enhancements in the CFSI and CCI, were observed at the MK and NF farms following the introduction of biosensors. Farm owners recognized the technology’s cost-effectiveness for heat detection, despite facing language barriers with some sensor brands. However, the study’s limited sample size limits its broader applicability, and concerns about data privacy and the complexity of the technology may impede wider adoption. Future research should tackle these challenges by developing new data-sharing methods, offering cost-effective monitoring solutions, and exploring cultural factors that influence technology adoption to build trust and promote the expansion of smart-sensing technologies in the dairy industry.

## Authors’ Contributions

JK, JL, PK, TS, DM, and CI: Conceptualization. PK and CI: Methodology. CI: Software and project administration, resources. JK, JL, NK, KP, PK, and CI: Investigation and formal analysis. PK, TS, and CI: Validation. JK, JL, NK, AR, and KP: Data curation and writing – original draft preparation. TS, DM, AR, and CI: Writing – review and editing. PK and CI: Visualization. TS and CI: Supervision. All authors have read, reviewed, and approved the final manuscript.
